# Stage at presentation of breast cancer in Luanda, Angola - a retrospective study

**DOI:** 10.1186/s12913-015-1092-9

**Published:** 2015-10-15

**Authors:** Lygia Vieira Lopes, Fernando Miguel, Helga Freitas, António Tavares, Salvador Pangui, Clara Castro, Gonçalo Forjaz Lacerda, Adhemar Longatto-Filho, Elisabete Weiderpass, Lúcio Lara Santos

**Affiliations:** Sagrada Esperança Clinic, Luanda, Angola; Angolan Institute of Cancer Control, Luanda, Angola; National Public Health Department, Ministry of Health, Luanda, Angola; Azores Cancer Registry, Azores Oncological Centre, Angra do Heroísmo, Portugal; Life and Health Sciences Research Institute (ICVS), School of Health Sciences, University of Minho, Braga, Portugal; Laboratory of Medical Investigation (LIM 14), Faculty of Medicine, São Paulo State University, São Paulo, Brazil; Molecular Oncology Research Center “Centro de Pesquisa em Oncologia Molecular” (CPOM), Barretos, Brazil; Cancer Registry of Norway, Oslo, Norway; Department of Community Medicine, Faculty of Health Sciences, University of Tromsø, The Arctic University of Norway, Tromsø, Norway; Department of Medical Epidemiology and Biostatistics, Karolinska Institutet, Stockholm, Sweden; Department of Genetic Epidemiology, Folkhälsan Research Center, Samfundet Folkhälsan, Helsinki, Finland; ONCOCIR - Education and Care in Oncology, Luanda, Angola; Experimental Pathology and Therapeutics Research Center, Portuguese Oncology Institute of Porto, Porto, Portugal; Department of Epidemiology, Portuguese Oncology Institute of Porto, Porto, Portugal

**Keywords:** Breast cancer, Angola, Clinical and pathological characterization

## Abstract

**Background:**

It is expected that, by 2020, 15 million new cases of cancer will occur every year in the world, one million of them in Africa. Knowledge of cancer trends in African countries is far from adequate, and improvements in cancer prevention efforts are urgently needed. The aim of this study was to characterize breast cancer clinically and pathologically at presentation in Luanda, Angola; we additionally provide quality information that will be useful for breast cancer care planning in the country_._

**Methods:**

Data on breast cancer cases were retrieved from the Angolan Institute of Cancer Control, from 2006 to 2014. For women diagnosed in 2009 (5-years of follow-up), demographic, clinical and pathological information, at presentation, was collected, namely age at diagnosis, parity, methods used for pathological diagnoses, tumor pathological characteristics, stage of disease and treatment. Descriptive statistics were performed.

**Results:**

The median age of women diagnosed with breast cancer in 2009 was 47 years old (range 25–89). The most frequent clinical presentation was breast swelling with axillary lymph nodes metastasis (44.9 %), followed by a mass larger than 5 cm (14.2 %) and lump (12.9 %). Invasive ductal carcinoma was the main histologic type (81.8 %). Only 10.1 % of cancer cases had a well differentiated histological grade. Cancers were diagnosed mostly at advanced stages (66.7 % in stage III and 11.1 % in stage IV).

**Discussion:**

In this study, breast cancer was diagnosed at a very advanced stage. Although it reports data from a single cancer center in Luanda, Angola it reinforces the need for early diagnosis and increasing awareness. According to the main challenges related to breast cancer diagnosis and treatment herein presented, we propose a realistic framework that would allow for the implementation of a breast cancer care program, built under a strong network based on cooperation, teaching, audit, good practices and the organization of health services.

**Conclusion:**

Angola needs urgently a program for early diagnosis of breast cancer.

## Background

It is expected that by 2020, 15 million new cases of cancer will occur every year in the world, one million of them in Africa. Knowledge of cancer trends in Africa is far from adequate, and all efforts to develop population based cancer registries are needed to obtain reliable data to guide Public Health authorities in planning and implementing cancer control programs, aiming to reduce cancer incidence, and related morbidity and mortality [1].

The cancer burden in Africa, including Angola, is likely to increase in the forthcoming decades, due to the increasing life expectancy of the population, changes in lifestyles associated with economic development, and longer survival of HIV patients receiving antiretroviral therapy, as HIV/AIDS patients have a substantially higher risk of developing cancer than the general population. The control of communicable diseases is certainly very important, but improvements of primary, secondary, and tertiary prevention efforts to deal with non-communicable diseases, including cancer, are urgently needed [2].

Angola has merely 3,541 doctors, 34,300 nurses and 6,414 health technicians for around 24.3 million people (48 % males and 52 % females), with a life expectancy at birth of 52 years. There are seven medical schools and several professional schools for nurses and other health professionals [3]. About 12 pathologists currently work in Angola. According to Trading Economics (Angola/indicators) and International Monetary Fund, the gross domestic product (GDP) in Angola was worth 124.18 billion USD in 2013, although it is largely dependent on oil price [4]. The Government has recently decided a new National Cancer Plan, which is a visionary and ambitious initiative to improve cancer control in the country [5].

The Angolan Institute of Cancer Control (IACC), former National Oncology Center of Luanda, is the oldest public center for the treatment of cancer patients in Angola. It has chemotherapy and radiotherapy facilities, diagnostic capabilities and experienced professionals. The Sagrada Esperança Clinic belongs to a public company named Endiama. This clinic has pathology facilities and a unit for the diagnosis, treatment and monitoring of breast diseases, with multidisciplinary treatment decisions implemented. At present, the clinic is organizing a chemotherapy service, as well as a unit for control of chronic pain and a breast cancer screening program for Endiama employees. The Girassol Clinic belongs to the public company Sonangol. This clinic has the capacity for diagnostic imaging, as well as pathological facilities. It also has a specifically built oncology service, with a dedicated ward, an outpatient hospital, a radiotherapy unit, a chemotherapy unit and Nuclear Medicine resources. The Américo Boavida Hospital, the David Bernardino Pediatric Hospital and the Josina Machel Hospital are all teaching hospitals where most of the cancer patients are diagnosed. These hospitals are able to perform pathological diagnosis by histology or cytology [6].

Breast cancer is the second most common cancer among women in Sub-Saharan Africa [7]. According to the International Agency for Research on Cancer (GLOBOCAN), the estimated breast cancer incidence rate in 2012 in Angola was 23.5 per 100 000 women, and the mortality rate was 11.7 per 100 000 [8]. In Angola, amongst the general population and university students, awareness and knowledge about breast cancer are extremely limited [9]. Our study aims to characterize breast cancer clinically and pathologically at presentation in Luanda, Angola; we additionally provide quality information that will be useful for breast cancer care planning in the country.

## Methods

### Study design, setting and data collections

Records of the Angolan Institute of Cancer Control, from 2006 to 2014, were reviewed by trained doctors, and information on the number of breast cancer cases was abstracted using a standard form.

In order to study a cohort of patients with a follow-up of 5 years, we decided to study all patients admitted and treated in the year 2009. We further abstracted more detailed information for this subset of patients, including data on age at diagnosis, parity, methods used for pathological diagnoses, tumor pathological characteristics, stage of disease, and treatment. Breast tumors were classified according to the 6th edition of TNM classification, which is based on the size of the primary tumor and presence of metastatic regional lymph nodes and/or of distant metastases [10]. Physical breast examination, mammography and/or breast ultrasound examination and fine-needle aspiration (FNA) are the commonly used diagnostic methods. Histology and FNA slides were reviewed by a pathologist according to the WHO classification [11].

### Statistical analysis

Descriptive statistics are given as frequencies, median, mean, minimum and maximum for continuous variables and as percentages for categorical variables. Kruskal-Wallis test was used to evaluate the differences in the median age according to stage for cases admitted and treated in 2009. Statistical analyses were performed using PASW Statistics for Windows, Version 18.0, 2009. Chicago: SPSS Inc. ®.

### Ethics

Permission to carry out this study was obtained by the Angolan Ministry of Health and corresponding ethics committee.

## Results

**Fig. 1:**
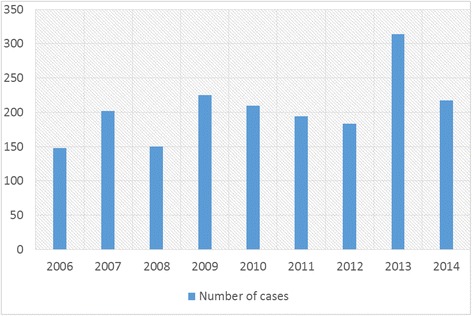
Number of breast cancer cases admitted and treated per year at IACC

From 2006 to 2014 there were 1,843 women admitted and treated with a diagnosis of breast cancer (Fig. 1). The median age at diagnosis was 47 years (16–87 years). The following distribution of the stage of disease was found: stage 0–2.1 %; stage I - 3.5 %; stage II - 12.2 %; stage III - 54.2 %; stage IV - 1.9 %; unclassified - 26.1 % (Table 1). Since no complete information on stage was available for the entire period, we could not evaluate if there were significant differences in the median age according to stage. However, using only data from 2009, no significant differences were found (*p* = 0.1). Invasive ductal cancer was the most common histologic type of breast cancer found in the whole series.

**Table 1:** Age (years) distribution according to stage of the disease

Stage	n (%)	Mean	Median	Minimum	Maximum
0	38 (2.1)	45.5	46	25	75
I	65 (3.5)	49.2	49	20	84
II	225 (12.2)	48.4	47	24	81
III	998 (54.2)	47.3	45	16	87
IV	35 (1.9)	44.1	44	23	60
unclassified	482 (26.1)	50.1	49	18	85

In 2009, a total of 225 women were admitted for treatment. Table 2 shows the women’ demographic, clinical and breast cancer information for year 2009. The median age of these women was 47 years (25–89 years); the majority of them were multiparous (72 %). Complete replacement of breast tissue, nipple retraction and deviation, oedema and ulceration of overlying skin, breast swelling with axillary nodal involvement were the most frequent clinical presentation. Invasive ductal carcinoma was the predominant histological type (81.8 %). Of all breast cancers diagnosed in 2009, 25.2 % were grade III (poorly-differentiated) tumors. FNA was performed in all cases and 37 % of these were histologically confirmed. All surgical specimens were histologically examined. An IHC reaction to define the molecular profile was only performed in one patient of this series and for this reason this variable was not considered for complementary analyses. Among the 225 cases, 176 (77.8 %) were classified as stages III and IV. Follow-up data was available only in 47.1 % (*n* = 106) of patients studied, with a median follow-up of 13.4 months (1–53.9 months). Consequently, no overall survival was calculated.

**Table 2:** Demographic, clinical and pathological figures for women included in study

Characteristics	n (%)
Age (years)	
20-29	10 (4.4)
30-39	37 (16.4)
40-49	91 (40,4)
50-59	47 (20.9)
60-69	23 (10.2)
70-79	12 (5.3)
80-89	5 (2.2)
Median	47 (range 25–89)
Parity	
Nulliparous	7 (3.1)
Multiparous	162 (72)
Without information	56 (24.9)
Clinical presentation	
Mammograms alteration	8 (3.6)
Lump (<5 cm)	29 (12.9)
Mass (>5 cm)	32 (14.2)
Nipple or skin retraction	21(9.3)
Ulcer	21(9.3)
Swelling and redness (inflammatory)	13 (5.8)
Breast swelling with axillar lymph nodes	101 (44.9)
Histhological Type	
Ductal carcinoma in situ	2 (0.9)
Invasive ductal carcinoma	184 (81.8)
Invasive lobullar carcinoma	2 (0.9)
Medullary	9 (4)
Papillary	17 (7.6)
Squamous cell carcinoma	1 (0.4)
Mucinous	1 (0.4)
Sarcoma	1 (0.4)
No classified	8 (3.6)
Grade	
I	45 (10.1)
II	123 (54.7)
III	57 (25.2)
Stage	
0	2 (0.9)
I	4 (1.8)
II	44 (19.5)
III	150 (66.7)
IV	25 (11.1)

Modified radical mastectomy was performed in 152 patients (67.5 %), and neo-adjuvant chemotherapy was given to 104 patients (46.2 %); adjuvant or palliative chemotherapy was given to the remaining patients. The most frequently used drugs were doxorubicin, cyclophosphamide, paclitaxel, methotrexate, 5-FU, cisplatin, tamoxifen and bisphosphonates. Less than 10 % of the patients of this series (2009) had radiotherapy.

## Discussion

This is the first paper characterizing the stage of breast cancer in Luanda, Angola at presentation. We only had access to data from a tertiary hospital in the capital city, Luanda, and therefore our data cannot be considered as representative of the true epidemiological pattern in Angola, where many women with breast lesions, in particular in remote areas, may never reach a health care facility. According to our findings, breast cancer diagnoses were performed mostly in multiparous young pre-menopause women, as it has been reported in others African studies [12, 13].

Breast cancer diagnosis was performed using physical examination, ultrasound and radiological examinations, and FNA. The modified triple test score (MTTS), which is an integration of clinical breast examination, ultrasound and FNA, may be introduced, since the MTTS showed 100 % diagnostic accuracy for breast cancer malignancy in women under age 40 [14].

The exposure and outcome information in a cohort study were identified retrospectively by using administrative datasets and by reviewing patient charts, but these are sometimes frail. Thus, the major limitation of this study is the lack of information (missing data) in the aforementioned datasets, which limits data analysis and interpretation. Although we do not have complete information on overall survival, given that breast cancer was mostly diagnosed at advanced stages, prognosis is expected to be very poor.

Mastectomy is the most widely used procedure for treatment of breast cancer, but in this particular context even well conducted surgeries have limited levels of accomplishment. No detailed information on tumour size could be obtained, but lymph node metastases were found mostly in the group of patients with a mass over 5 cm, nipple or skin retraction and skin ulceration. This is in accordance with other studies, which have found a significant association between lymph node metastasis and tumour size [15].

Additionally, neo-adjuvant therapy is frequently used to treat patients with locally advanced breast cancer, but generally have little influence in the patient outcome. A similar disturbing scenario was also described in Nigeria [16].

Recently, after 2009, the National Oncological Centre (IACC) and the private Girassol Clinic in Luanda have installed radiotherapy facilities and recruited radiotherapists for working on a full-time basis, which is a promising advantage for breast cancer treatment. Currently, the patients treated with radiation at IACC and Girassol Clinic include breast cancer patients [17].

Accumulating molecular data may enable more accurate diagnoses and support therapeutic decisions. It is well established that according to the molecular profile of breast cancer, translated by IHC characterization, a panel of therapeutic actions should be addressed to minimize the impact of the disease [18].

A pro-active program for early detection of breast cancer and the complete characterization of the tumours should be implemented in an Angola breast cancer programme [19]. This country does not have the infrastructural capacity in terms of health care facilities, professionals and budget to implement and maintain a program for a population-based screening programme.

Reliable statistics of cancer incidence and mortality in Angola are not presently available. To allow for a proper planning of both secondary and tertiary preventable actions for breast cancer, by properly planning health care services, such statistics are needed. As suggested by Harford et al., twining between countries with medium/high income and low/middle income would allow the sharing of knowledge and experience in cancer registration best practices and tools for breast (and other cancer types) diagnosis and treatment [20]. Thus, cooperation between a well functioning and established cancer registry such as the Azores Cancer registry (Portugal) and the African Cancer Registry Network (AFCRN) would facilitate the establishment of a population-based cancer registry - where all new cancer cases occurring in a defined population are recorded - in Luanda. Hospital-based registries can be the first important step into the establishment of such a population-based cancer registry. Moreover, collaboration enhances the potential of both systems, especially in developing countries [21]. To accomplish this, Angolan National Centre of Oncology (IACC) has recently organized and sponsored a course entitled ‘Cancer Registration – Principles and Methods’.

It is also impossible to ignore the impact that ultrasound equipment has made within medical education. Ultrasound has played an essential role in point-of-care of breast cancer diagnostics, and implementing ultrasound training into medical education is the next logical step in our breast cancer program.

**Fig. 2:**
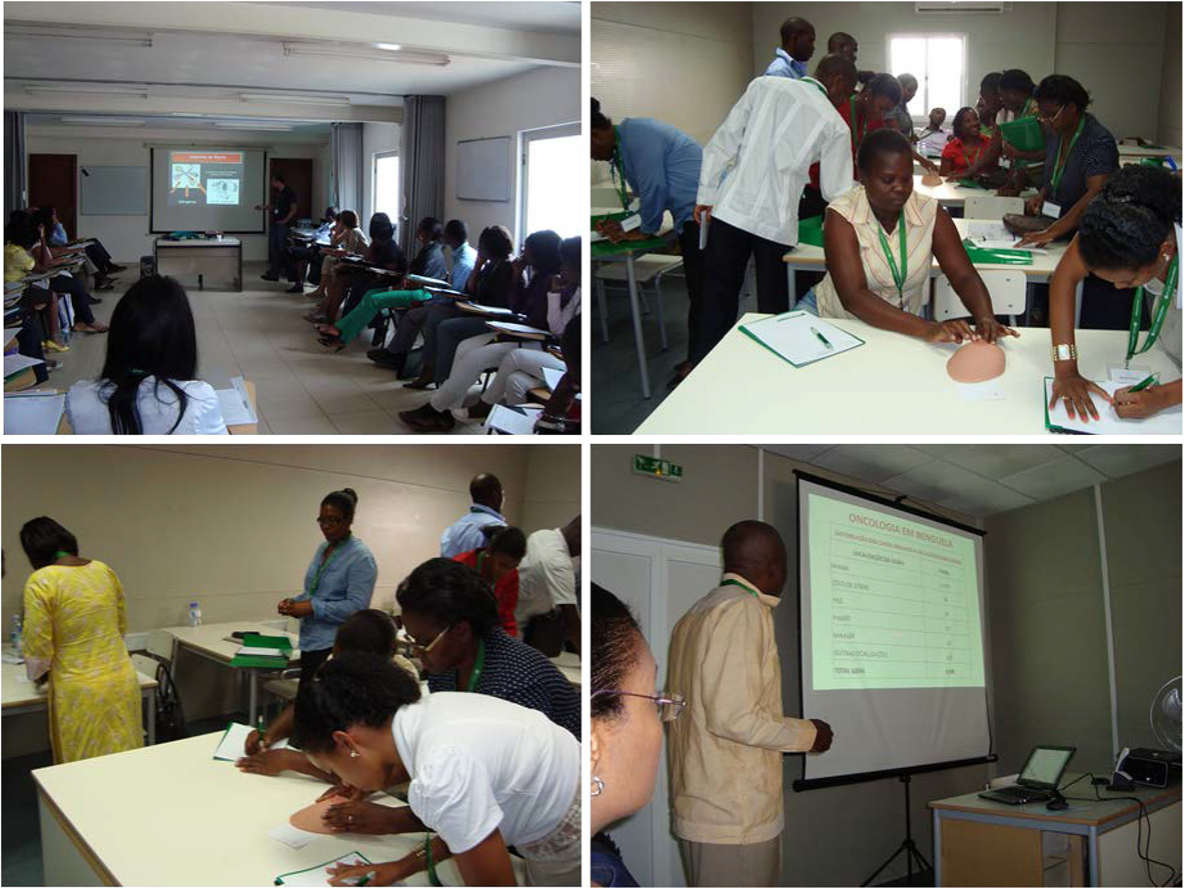
Medical education of general doctors of municipal hospitals regarding breast cancer. Specific consent to publish the images was obtained from all individuals

“Municipalities against cancer” is a comprehensive educational program that includes general doctors, nurses and teachers; it was created to discuss and plan strategies for cancer control of cervix, breast and prostate malignancies and patient management under the Angola Municipal Health Services Strengthening Project. The aims of this program are health education, early breast cancer diagnosis and patient management (Fig. 2). The Breast Health Global Initiative recommendations from the 2007 Summit were to promote breast self-awareness and clinical breast examination (CBE) at the basic level and to encourage women to seek medical evaluation of breast problems and diagnostic imaging, such as ultrasound and mammography, for suspicious breast nodules [22]. Ultrasound is superior and a cost effective alternative for the assessment of the symptomatic young patient and is an optimal modality for imaging guidance to improve accuracy of fine-needle aspiration [23]. General practitioners and medical students should be offered training to learn how to use this diagnostic tool.

Management of breast cancer requires an extensive and urgent approach that combines actions such as: effective prevention, early diagnosis, surgical therapies and timely access to cost-effective chemotherapy or radiation therapy, as well as palliative care. Availability and affordability of anticancer medicines is another important step in this fight. Efforts should be made to ensure adequate, cheap and uninterrupted supply of anticancer medicines, and radiation treatments to breast cancer patients. Additionally, molecular characterization of these neoplasms is fundamental since this may condition the therapeutic profile. However, a successful management of breast cancer also requires specialized oncology-trained doctors (surgeons, medical oncologist and radiotherapists), nurses, imaging technicians and pathologists in order to develop a proficient oncologic care and a multidisciplinary approach (Table 3) [24].

**Table 3:** Urgent actions needed for breast cancer program in Angola

Task	Actions
Structural developments	Hospital-based cancer registries, as a crucial step in order to establish a population-based cancer registry in Luanda.
Capacity building and awareness	“Municipalities against cancer” a comprehensive educational program that includes general doctors, nurses and teachers.
Diagnosis	Early detection;
	Proactive programme for early detection that includes clinical and ultrasound examination by municipal doctors. Introduction of the modified triple test score (MTTS);
	Tissue procurement (FNA and histology);
	Pathology diagnosis accuracy and determination of ER, PR, HER2 and KI-67 (protein) status by IHC.
Treatment decision	Multidisciplinary integrated treatment team in order to provide the most comprehensive treatment plan.
Adequate treatment	Surgery: The best surgical treatment (Surgical oncology training);
	Systemic therapy: drugs availability, affordability and uninterrupted supply, safe storage and preparation, adequate prescription and administration, management of side effects;
	Radiation therapy: to be included in breast treatment protocol, management of side effects related to radiation;
	Accessibility to cost-effective chemotherapy or radiation therapy, and palliative care.
Follow-up	Shared and supported follow-up program including all levels of care and adequate registration of follow-up data.
Palliative care	Pain control and adequate management of end-of-life care.

*ER* estrogen receptor, *PR* progesterone receptor, *IHC* immunohistochemistry, *HER2* human epidermal growth factor receptor, *KI-67 (protein)* proliferation marker

## Conclusion

In Luanda, Angola, breast cancer is common. Most cases are diagnosed at an advanced stage, which predicts a poor prognosis. Therefore, Angola needs a program for early diagnosis of breast cancer. Clinical breast examination, ultrasound and/or mammography and FNA may be useful in this context. Radiotherapy is one of the fundamental treatments at these stages and should be widely used. The characterization of IHC/molecular breast cancer subtypes is urgent, in order to achieve the best systemic treatment approach.

## References

[1] O’Brien KS, Soliman AS, Awuah B, Jiggae E, Osei-Bonsu E, Quayson S, Adjei E, Thaivalappil SS, Abantanga F, Merajver SD (2013). Establishing effective registration systems in resource-limited settings: cancer registration in kumasi Ghana. J Registry Manag.

[2] Soerjomataram I, Lortet-Tieulent J, Parkin DM, Ferlay J, Mathers C, Forman D, Bray F (2012). Global burden of cancer in 2008: a systematic analysis of disability-adjusted life-years in 12 world regions. Lancet.

[3] Fronteira I, Sidat M, Fresta M, Sambo Mdo R, Belo C, Kahuli C, Rodrigues MA, Ferrinho P (2014). The rise of medical training in Portuguese speaking African countries. Hum Resour Health.

[4] International Monetary Fund (Publication Services). Technical assistant report –Angola – fuel price subsidy reform the way forward. Washington, D.C. 2015. http://www.imf.org/external/pubs/ft/scr/2015/cr1528.pdf. Accessed 8 Fev 2015.

[5] USAID. National Plan for Health Development 2012–2015. https://www.hfgproject.org/angolas-moh-embraces-stronger-governance/.(2015) Accessed 8 Fev 2015.

[6] Lopes LV, Conceição AV, Oliveira JB, Tavares A, Domingos C, Santos LL (2012). Cancer in Angola, resources and strategy for its control. Pan Afr Med J.

[7] Jemal A, Bray F, Center MM, Ferlay J, Ward E, Forman D (2011). Global cancer statistics. CA Cancer J Clin.

[8] Ferlay J, Soerjomataram I, Ervik M, Dikshit R, Eser S, Mathers C, Rebelo M, Parkin DM, Forman D, Bray F (2013). LOBOCAN 2012 v1.0, Cancer Incidence and Mortality Worldwide: IARC CancerBase No. 11 [Internet].

[9] Sambanje MN, Mafuvadze B (2012). Breast cancer knowledge and awareness among university students in Angola. Pan Afr Med J.

[10] Singletary SE, Greene FL, Breast Task Force (2003). Revision of breast cancer staging: the 6th edition of the TNM Classification. Semin Surg Oncol.

[11] World Health Organization (2003). Tumours of the Breast and Female Genital Organs.

[12] Mody GN, Nduaguba A, Ntirenganya F, Riviello R (2013). Characteristics and presentation of patients with breast cancer in Rwanda. Am J Surg.

[13] Sighoko D, Kamaté B, Traore C, Mallé B, Coulibaly B, Karidiatou A, Diallo C, Bah E, McCormack V, Muwonge R, Bourgeois D, Gormally E, Curado MP, Bayo S, Hainaut P (2013). Breast cancer in pre-menopausal women in West Africa: analysis of temporal trends and evaluation of risk factors associated with reproductive life. Breast.

[14] Morris KT, Vetto JT, Petty JK, Lum SS, Schmidt WA, Toth-Fejel S, Pommier RF (2002). A new score for the evaluation of palpable breast masses in women under age 40. Am J Surg.

[15] Wiggett WS, Louw M, Karusseit VO (2012). The histology of peau d’orange in breast cancer - what are the implications for surgery?. S Afr J Surg.

[16] T P Kingham, O I Alatise, V Vanderpuye, C Casper, FA Abantanga, T B Kamara, et al. Treatment of cancer in sub-Saharan Africa. Lancet Oncology 2013–04,14: e158-e167. 10.1016/S1470-2045(12)70472-2 23561747

[17] Fernando M, Conceição AV, Lopes LV, Bernardo D, Monteiro F, Bessa F, Santos C, Oliveira JB, Santos LL (2015). Establishing of cancer units in low or middle income African countries: Angolan experience - a preliminary report. Pan Afr Med J.

[18] Spitale A, Mazzola P, Soldini D, Mazzucchelli L, Bordoni A (2009). Breast cancer classification according to immunohistochemical markers: clinicopathologic features and short-term survival analysis in a population-based study from the South of Switzerland. Ann Oncol.

[19] López-Gómez M, Malmierca E, de Górgolas M, Casado E (2013). Cancer in developing countries: the next most preventable pandemic. The global problem of cancer. Crit Rev Oncol Hematol.

[20] Harford JB, Otero IV, Anderson BO, Cazap E, Gradishar WJ, Gralow JR, Kane GM, Niëns LM, Porter PL, Reeler AV, Rieger PT, Shockney LD, Shulman LN, Soldak T, Thomas DB, Thompson B, Winchester DP, Zelle SG, Badwe RA (2011). Problem solving for breast health care delivery in low and middle resource countries (LMCs): consensus statement from the Breast Health Global Initiative. Breast.

[21] Valsecchi MG, Steliarova-Foucher E (2008). Cancer registration in developing countries: luxury or necessity?. Lancet Oncol.

[22] Anderson BO, Yip CH, Smith RA, Shyyan R, Sener SF, Eniu A, Harford J (2008). Guideline implementation for breast healthcare in low‐income and middle‐income countries. Cancer.

[23] Yip C-H, Smith RA, Anderson BO, Miller AB, Thomas DB, Ang E-S, Caffarella RS, Corbex M, Kreps GL, McTiernan A, and on behalf of the Breast Health Global Initiative Early Detection Panel (2008). Guideline implementation for breast healthcare in low- and middle-income countries. Cancer.

[24] Shulman LN, Mpunga T, Tapela N, Wagner CM, Fadelu T, Binagwaho A (2014). Bringing cancer care to the poor: experiences from Rwanda. Nat Rev Cancer.

